# Characterization of Alzheimer’s Disease Using Ultra-high b-values Apparent Diffusion Coefficient and Diffusion Kurtosis Imaging

**DOI:** 10.14336/AD.2018.1129

**Published:** 2019-10-01

**Authors:** Yingnan Xue, Zhenhua Zhang, Caiyun Wen, Huiru Liu, Suyuan Wang, Jiance Li, Qichuan Zhuge, Weijian Chen, Qiong Ye

**Affiliations:** ^1^Department of Radiology, The First Affiliated Hospital of Wenzhou Medical University, Wenzhou, China; ^2^Zhejiang Provincial Key Laboratory of Aging and Neurological Disorder Research, Department of Neurosurgery, The First Affiliated Hospital of Wenzhou Medical University, Wenzhou, China

**Keywords:** Alzheimer’s Disease, Ultra-high B-values Apparent Diffusion Coefficient, Diffusion Kurtosis Imaging, Apparent Diffusion Coefficient, ADC_uh, DKI

## Abstract

The aim of the study is to investigate the diffusion characteristics of Alzheimer’s disease (AD) patients using an ultra-high b-values apparent diffusion coefficient (ADC_uh) and diffusion kurtosis imaging (DKI). A total of 31 AD patients and 20 healthy controls (HC) who underwent both MRI examination and clinical assessment were included in this study. Diffusion weighted imaging (DWI) was acquired with 14 b-values in the range of 0 and 5000 s/mm^2^. Diffusivity was analyzed in selected regions, including the amygdala (AMY), hippocampus (HIP), thalamus (THA), caudate (CAU), globus pallidus (GPA), lateral ventricles (LVe), white matter (WM) of the frontal lobe (FL), WM of the temporal lobe (TL), WM of the parietal lobe (PL) and centrum semiovale (CS). The mean, median, skewness and kurtosis of the conventional apparent diffusion coefficient (ADC), DKI (including two variables, D_app_ and K_app_) and ADC_uh values were calculated for these selected regions. Compared to the HC group, the ADC values of AD group were significantly higher in the right HIP and right PL (WM), while the ADC_uh values of the AD group increased significantly in the WM of the bilateral TL and right CS. In the AD group, the K_app_ values in the bilateral LVe, bilateral PL/left TL (WM) and right CS were lower than those in the HC group, while the D_app_ value of the right PL (WM) increased. The ADC_uh value of the right TL was negatively correlated with MMSE (mean, r=-0.420, p=0.019). The ADC value and D_app_ value have the same regions correlated with MMSE. Compared with the ADC_uh, combining ADC_uh and ADC parameters will result in a higher AUC (0.894, 95%CI=0.803-0.984, p=0.022). Comparing to ADC or DKI, ADC_uh has no significant difference in the detectability of AD, but ADC_uh can better reflect characteristic alternation in unconventional brain regions of AD patients.

Alzheimer’s disease (AD) is a progressive neuro-degenerative disease. As reported by the World Health Organization (WHO), the prevalence of dementia in the world is estimated to be 50 million, and there are nearly 10 million new cases every year, with AD potentially contributing to 60-70% of these cases [1, 2]. The pathogenesis of AD is extremely complicated and has never been clearly clarified. At present, many studies have shown that the deposition of β-amyloid peptide (Aβ) and neurofibrillary tangles (NFTs) are the main pathological changes in Alzheimer's disease [3-6], while apolipoprotein E4 (ApoE4), α-synuclein (α-Syn), aquaporin-4 (AQP4) and hyperphosphorylated tau play important roles in the process of Aβ deposition and NFTs [7-13].

Recently, it was reported that the herpesvirus may be the original reason for AD [14]. In this study, they found a high level of human herpesvirus (HHV-6A and HHV-7) in the brain regions that present AD neuropathological changes. Many studies have demonstrated the deposition of Aβ resistance to the herpesvirus infection. The herpesvirus can also induce the formation of Aβ deposits [15-18]. Regardless of the initial cause of AD, the abnormal deposition of Aβ is still an important step in the occurrence and development of AD [14, 19, 20].

The imbalance between the production and clearance of Aβ leads to the deposition of Aβ, resulting in increased soluble Aβ and increased plaque accumulation in the brain. AQP4 has been given more attention in recent years in the research of AD. AQP4 is an important carrier of water metabolism in the brain. Aβ in the brain can be cleaned by water transport, and a lack of AQP4 can decrease the clearance of soluble Aβ [9, 21]. A wealth of studies has shown that the expression and distribution of AQP4 are altered in clinical and animal AD models [22-24].

The ultra-high b-values apparent diffusion coefficient (ADC_uh) could eliminate the influence of microvascular perfusion and the signal intensity changes, which are mainly the result of the slow diffusion component [25]. Some scholars believe that ADC_uh reflects the transport of water via aquaporins, which might be linked to the expression of AQP4 [26, 27]. Compared to ADC, ADC_uh showed a relatively higher sensitivity to WM degeneration in AD [28].

Varied non-Gaussian diffusion models show potential in aiding in the understanding of microstructure alternations in AD. Moreover, compared with diffusion tensor imaging (DTI), diffusion kurtosis imaging (DKI) can reflect the microstructure changes of white fiber more accurately and sensitively [29-31]. Correlations between the microstructural alterations and severity of cognitive deficiency in AD were demonstrated using DKI [32].

The purpose of this study was to evaluate the diffusion characteristics of ADC_uh and DKI and to explore their role in the differential diagnosis of AD.

## MATERIALS AND METHODS

This study was approved by the institutional review board, and the consents were signed.

### Subjects

Thirty-one patients who were suspected of mild to moderate cognitive impairment were included in this study. All patients underwent the Mini-Mental State Examination (MMSE). Inclusion criteria were as follows: (1) Ages range, 50-85 (including 50 and 85 years); (2) Most likely diagnostic criteria for AD in accordance with the National Institute of Neurological and Communicative Disorders and Stroke (NINCDS) - Alzheimer's Disease and Related Disorders Association (ADRDA) (NINCDS-ADRDA) (1984); (3) MMSE Total score, 11 ≤ MMSE total score ≤ 26 (for primary school education level, 11≤ MMSE total score ≤ 22); (4) Hachinski ischemic scale (HIS), total score ≤ 4; Hamilton Depression Rating Scale (HAMD17), total score ≤ 10; (5) The patients' memory decline lasts at least 12 months and there is a trend of progressive aggravation; (6) For subjects ≤ 70 years old, the grade of white matter (WM) damage (Fazekas scale for WM lesion) ≤1; For subjects > 70 years old, grade of WM damage ≤2; (7) Lacunar infarct, diameter >2 cm, number of lesions less than or equal to 2; (8) Key areas such as the thalamus (THA), hippocampus (HIP), entorhinal cortex, paranasal cortex, and other cortical and subcortical nucleus clumps have no infraction, and an MRI showed the greatest possibility of AD; (9) Neurological examination had no obvious signs; (10)The patient has a degree of primary education or above, and has the ability to complete the program's cognitive ability tests and other tests; (11) Exclusion criteria: other types of dementia, history of nervous system disease, people with mental illness.

Twenty control subjects participated in the study, and all of them underwent the MMSE. Inclusion criteria were as follows: (1) Ages range, 50-85 (including 50 and 85 years); (2) The patient has a degree of primary education or above; grade of Fazekas scale for WM lesions ≤1 (mild WM lesions), lacunar infarct, diameter >2 cm, number of lesions were less than or equal to 2; (3) Key areas such as the THA, HIP, entorhinal cortex, paranasal cortex, other cortical and subcortical nucleus clumps have no infraction, no brain atrophy; (4) Neurological examination has no obvious sign; No obvious cognitive impairment; (5) Exclusion criteria: have suffered from nervous system disease, psychiatric patient, pressure≥100 mmHg or <60 mmHg, patients with unstable or severe heart/lung/liver/kidney/hematopoietic diseases.

Moreover, 10 healthy volunteers aged between 23 and 28 were recruited for a repeatability verification of our test. On the same day, each volunteer underwent an MRI scan twice, and the scanning instrument and scanning sequences were consistent with those of AD patients.

### Image acquisition and processing

All MR scans were conducted at a 3.0T Philips (Achieva, The Netherlands) system with an 8-channel receive-only head coil. Diffusion weighted imaging (DWI) data were acquired with a single-shot spin-echo echo planar imaging (EPI) sequence in the following parameters: echo time/repetition time (TE/TR) =113/8000 ms, field of view = 220*220 mm^2^, matrix=124*120, reconstruction = 256*256, slice thickness = 5.0 mm without gap, No. of slices = 25, SENSE = 2.0, 14 b-values = 0, 25, 50, 75, 100, 150, 200, 500, 800, 1000, 2000, 3000, 4000, 5000 s/mm^2^, scan time = 5 min 36 sec. Voxelwise-computed diffusion weighted imaging (vcDWI) was acquired to achieve an anatomical reference that was geometrically identical to the previously acquired DWI data.

**Figure 1.**
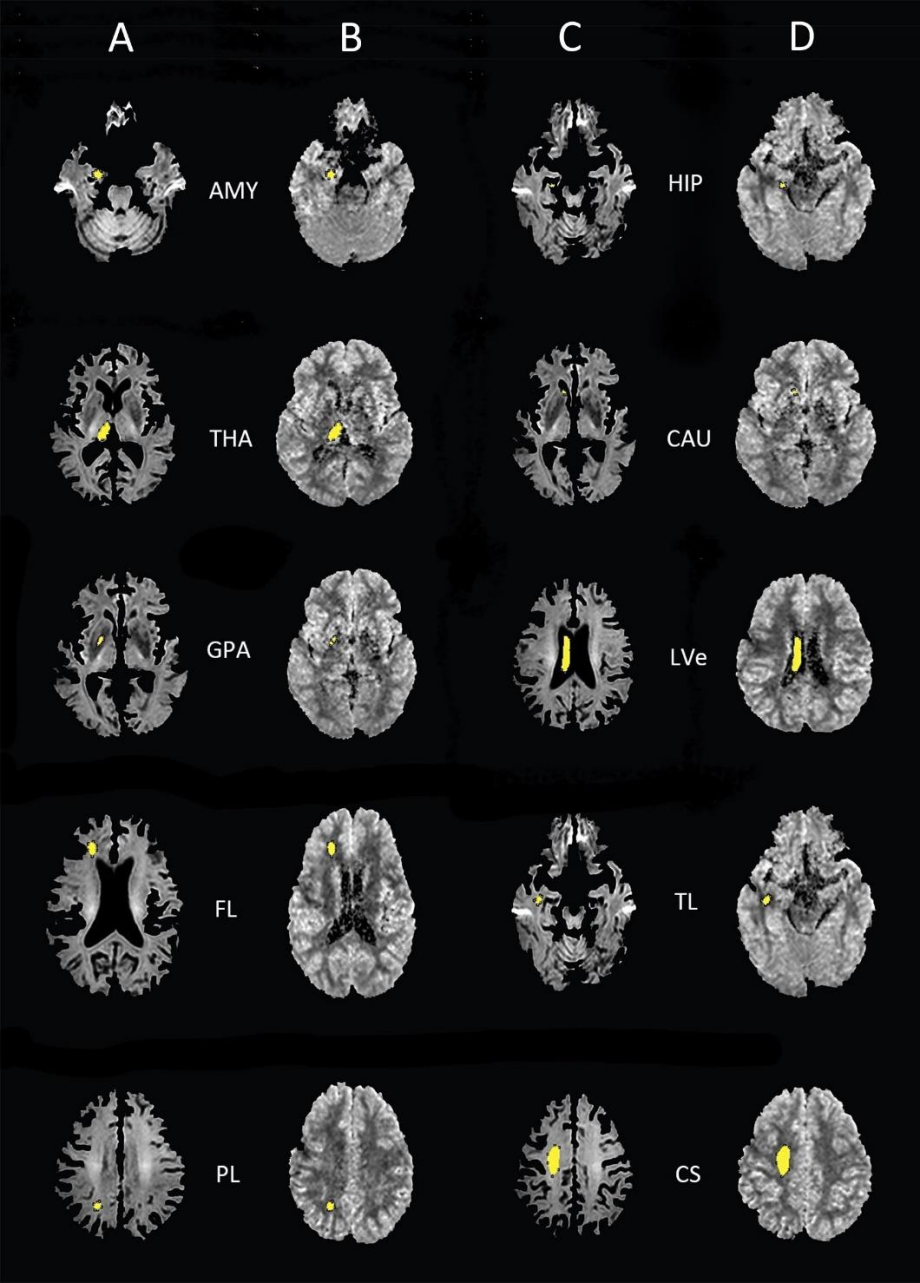
Selections of region of interest. (A, C) The selected ROIs on the vcDWI maps. (B, D) The ROIs were projected onto the ADC_uh maps. The yellow part is the ROI range. AMY, amygdala; HIP, hippocampus; THA, thalamus; CAU, caudate; GPA, globus pallidus; LVe, lateral ventricles; FL, frontal lobe (WM); TL, temporal lobe (WM); PL, parietal lobe (WM); CS, centrum semiovale.

The DWI data were preprocessed in FSL (Release 5.0, Oxford, UK) for the brain extraction and the motion correction. All parametric maps were generated by home-developed programming in MATLAB (The MathWorks Inc., Natick, MA, USA). ADC was calculated with mono-exponential fitting of signal intensities over b-values=0, 200, 500, 800, 1000 s/mm^2^. ADC_uh was calculated with mono-exponential fitting of signal intensities over b-values= 2000, 3000, 4000, 5000 s/mm^2^ [26]. For DKI, signal intensities all less than or equal to b-value= 3000 were used for fitting [33].

$$S=S_{0}e^{\left\{-bD_{app}+\frac{1}{6}b^{2}D_{app}{}^{2}K_{app}\right\}}$$

where S is the signal intensity, S_0_ is the signal intensity at b = 0, D_app_ is the diffusion coefficient, and K_app_ quantifies the deviation of the dispersion mode from the Gaussian distribution. The Levenberg-Marquardt (LM) algorithm was applied for optimization. Points with values < 0 are nulled.

vcDWI is voxelwise-computed DWI, and its maps can be calculated as follow [34]:

$$vcDWI=S_{0}e^{-ADC^{2}\cdot 10^{6}}$$

Regions of interest (ROIs) were manually drawn on vcDWI in ImageJ (NIH, USA). Twelve structures: bilateral amygdala (AMY), hippocampus (HIP), thalamus (THA), caudate (CAU), globus pallidus (GPA), lateral ventricles (LVe), WM of the frontal lobe (FL), WM of the temporal lobe (TL), WM of the parietal lobe (PL) and Centrum semiovale (CS), were analyzed. All ROIs were acquired by avoiding the boundary of the brain area in both vcDWI and ADC_uh maps. The outline of ROIs was performed by two radiologists in consensus (5 and 8 years of experience in neuroimaging diagnosis). Representative vcDWI and ADC_uh maps with ROIs are shown in Fig. 1.

### Statistical analysis

Data are presented in the form of mean ± STD. The mean, median, skewness, kurtosis of parameters (ADC, ADC_uh, D_app_, K_app_) and age, MMSE between AD and the control group were compared using the Student’s t test or the Mann-Whitney U test. The gender distributions of both groups were compared using the chi-square test. Correlations between the diffusion characteristics and MMSE were evaluated using the Spearman’s rank correlation, and this step was performed in SPSS (IBM Corp, version 25.0). Rejection of the parameters with a Coefficient of Variance (CV) is greater than 0.5. Binary logistic regression analyses (backward, wald) were used to select data (SPSS, IBM Corp, version 25.0). Receiver operating characteristic (ROC) was used to assess the diagnostic utility of ADC_uh and DKI parameters. All classification analyses and evaluations were implemented in MedCalc (version 18.6). P values < 0.05 were considered statistically significant.

All data of repeatability experiments were categorized by parameters, and Bland-Altman analyses were used to evaluate the consistency of the two tests, and Spearman’s rank correlation was used to evaluate the correlation between the two tests.

**Table 1:** Demographic and cognitive characteristics of all participants.

	AD	HC	*p*-value
Number (M/F)	11/20	8/12	0.774^*^
Age(years)	64.94±8.205	56.70±6.258	0.000^**^
MMSE	18.48±4.711	27.85±1.565	0.000^**^

* Chi-square test;

** Unpaired t-test, two-tailed test; MMSE=Mini-Mental State Examination, AD = Alzheimer's disease; HC = Healthy control.

## RESULTS

The subject’s clinical and neuropsychological data are summarized in Table 1. The HC group has lower MMSE scores than the AD group (AD: 18.48±4.711; HC: 27.85±1.565; P< 0.05) as expected. The mean age of the HC group (56.70±6.26 years) was 8.24 years less than the AD group (64.94±8.21 years). There was no significant difference in gender composition between these two groups.

**Figure 2.**
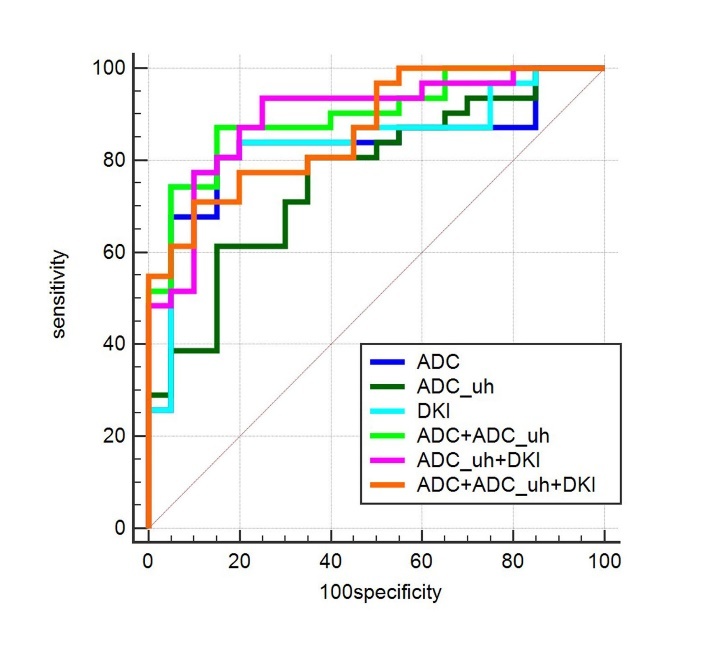
Receiver-operating characteristic curves (ROC) of classifications between AD and HC patients. ADC, ADC_uh, and DKI were separately assessed for differential diagnosis. Then, any combination of them was assessed separately. Finally, all of them was assessed together. Compared to ADC_uh, a higher AUC was obtained by combining ADC_uh values and ADC values (0.897, 95% CI=0.779-0.964, p=0.022). There was no significant difference between the other ROCs. The diagonal line represents a random classification performance.

**Table 2:** Comparisons of regional diffusion intensity in ADC or ADC_uh between AD and HC group.

		Mean ± SD	CV	P-value
ADC				
right HIP _mean_ (×10^-3^mm/s)	AD	0.961±0.126	0.131	0.008
	HC	0.874±0.095	0.109	
right HIP _median_ (×10^-3^mm/s)	AD	0.956±0.116	0.122	0.001
	HC	0.877±0.095	0.109	
left CAU _skewness_	AD	0.053±0.554	10.484	0.013
	HC	-0.313±0.515	-1.645	
right LVe_ skewness_	AD	0.377±0.881	2.338	0.000
	HC	-0.619±0.873	-1.410	
left FL _kurtosis_	AD	2.888±0.689	0.239	0.036
	HC	0.254±0.513	0.202	
right PL _mean_ (×10^-3^mm/s)	AD	0.815±0.091	0.111	0.002
	HC	0.750±0.051	0.068	
right PL _median_ (×10^-3^mm/s)	AD	0.818±0.094	0.115	0.003
	HC	0.754±0.050	0.066	
ADC_uh				
left THA _mean_ (×10^-3^mm/s)	AD	0.358±0.032	0.089	0.047
	HC	0.336±0.046	0.123	
left LVe _kurtosis_	AD	3.16±0.800	0.253	0.028
	HC	2.71±0.608	0.224	
right TL _mean_ (×10^-3^mm/s)	AD	0.274±0.042	0.154	0.033
	HC	0.252±0.029	0.116	
right TL _median_ (×10^-3^mm/s)	AD	0.273±0.045	0.165	0.032
	HC	0.249±0.030	0.120	
left TL _mean_ (×10^-3^mm/s)	AD	0.276±0.039	0.141	0.022
	HC	0.250±0.038	0.152	
left TL _median_ (×10^-3^mm/s)	AD	0.273±0.038	0.138	0.038
	HC	0.249±0.042	0.170	
right CS _mean_ (×10^-3^mm/s)	AD	0.273±0.038	0.088	0.021
	HC	0.203±0.015	0.073	
right CS _median_ (×10^-3^mm/s)	AD	0.214±0.019	0.089	0.016
	HC	0.201±0.015	0.074	

HIP, Hippocampus; THA, Thalamus; CAU, Caudate nucleus; LVe, Lateral ventricle; FL, Frontal lobe; TL, Temporal lobe; PL, Parietal lobe; CS, Centrum semiovale. P-value < 0.05 for all.

As shown in Table 2, the ADC values of the right HIP and the right PL WM of the AD group were higher than the HC group (mean and median, p < 0.05 for all). The ADC value of the left FL was higher than that of HC (kurtosis, p= 0.036). The ADC values of the left CAU and the right LVe were all higher than those of HC (skewness, p < 0.05 for all). The ADC_uh values of the bilateral TL WM and right CS of the AD were higher than those of the HC (mean and median, p<0.05 for all). The ADC_uh value of the left THA was higher in the AD group (mean, p=0.047), and the ADC_uh value of left LVe was higher than that of HC (Kurtosis, p=0.028).

D_app_ values of the right PL (mean and median) and left FL (Kurtosis) were significantly higher than those of the HC group (p<0.05), and K_app_ values in the bilateral PL WM, left LVe WM, left TL WM, and right CS showed obvious differences with the HC group (mean and median of K_app_, p<0.05). The right THA (median of K_app_), right LVe (mean of K_app_) and boilateral FL (kurtosis of K_app_) presented significant differences between these two groups (p<0.05). All of the above parameters are listed in Table 3.

For AD patients, right PL values in the ADC map and D_app_ map (mean and median) were negatively correlated with MMSE, while the values of ADC_uh in the right TL were negatively correlated with MMSE, as listed in Table 4. The kurtosis of the ADC and D_app_ values from the left FL were significantly positively correlated with MMSE (rho=0.550, p=0.001; rho=0.546, p=0.001, respectively). Moreover, the combination of ADC_uh and ADC values lead to a higher AUC (0.897, 95%CI= 0.779-0.964, p= 0.022) compared to only the ADC_uh values (Table 5).

**Table 3:** Comparisons of regional diffusion intensities in D_app_ or K_app_ between the AD and HC groups.

		Mean±SD	CV	P-value
D_APP_				
left FL _kurtosis_	AD	3.020±0.775	0.257	0.030
	HC	2.620±0.740	0.282	
right PL _mean_ (×10^-3^mm/s)	AD	0.942±0.105	0.112	0.003
	HC	0.870±0.060	0.069	
right PL _median_ (×10-3mm/s)	AD	0.943±0.107	0.113	0.004
	HC	0.870±0.063	0.073	
K_APP_				
right THA _median_	AD	0.713±0.072	0.101	0.046
	HC	0.754±0.075	0.099	
right LVe _mean_	AD	0.266±0.049	0.185	0.042
	HC	0.296±0.069	0.233	
left LVe_ mean_	AD	0.263±0.049	0.187	0.004
	HC	0.298±0.044	0.149	
left LVe_ median_	AD	0.279±0.040	0.143	0.006
	HC	0.308±0.038	0.125	
right FL _kurtosis_	AD	5.950±3.140	0.573	0.009
	HC	3.940±2.460	0.624	
left FL _kurtosis_	AD	5.750±3.010	0.524	0.019
	HC	4.380±3.250	0.742	
left TL _mean_	AD	0.740±0.106	0.144	0.009
	HC	0.816±0.090	0.111	
left TL _median_	AD	0.754±0.104	0.138	0.018
	HC	0.819±0.086	0.105	
right PL _mean_	AD	1.000±0.139	0.138	0.019
	HC	1.090±0.128	0.117	
right PL _median_	AD	0.994±0.127	0.128	0.015
	HC	1.090±0.133	0.122	
left PL _mean_	AD	0.949±0.146	0.154	0.007
	HC	1.070±0.142	0.133	
left PL _median_	AD	0.940±0.143	0.152	0.010
	HC	1.050±0.146	0.139	
right CS _mean_	AD	1.060±0.113	0.107	0.011
	HC	1.150±0.102	0.089	
right CS _median_	AD	1.040±0.109	0.105	0.004
	HC	1.130±0.104	0.092	

D_app_ is the diffusion coefficient (unit: ×10-3mm2/s); K_app_ quantifies the deviation of the dispersion mode from the Gaussian distribution (unitless).

**Figure 3.**
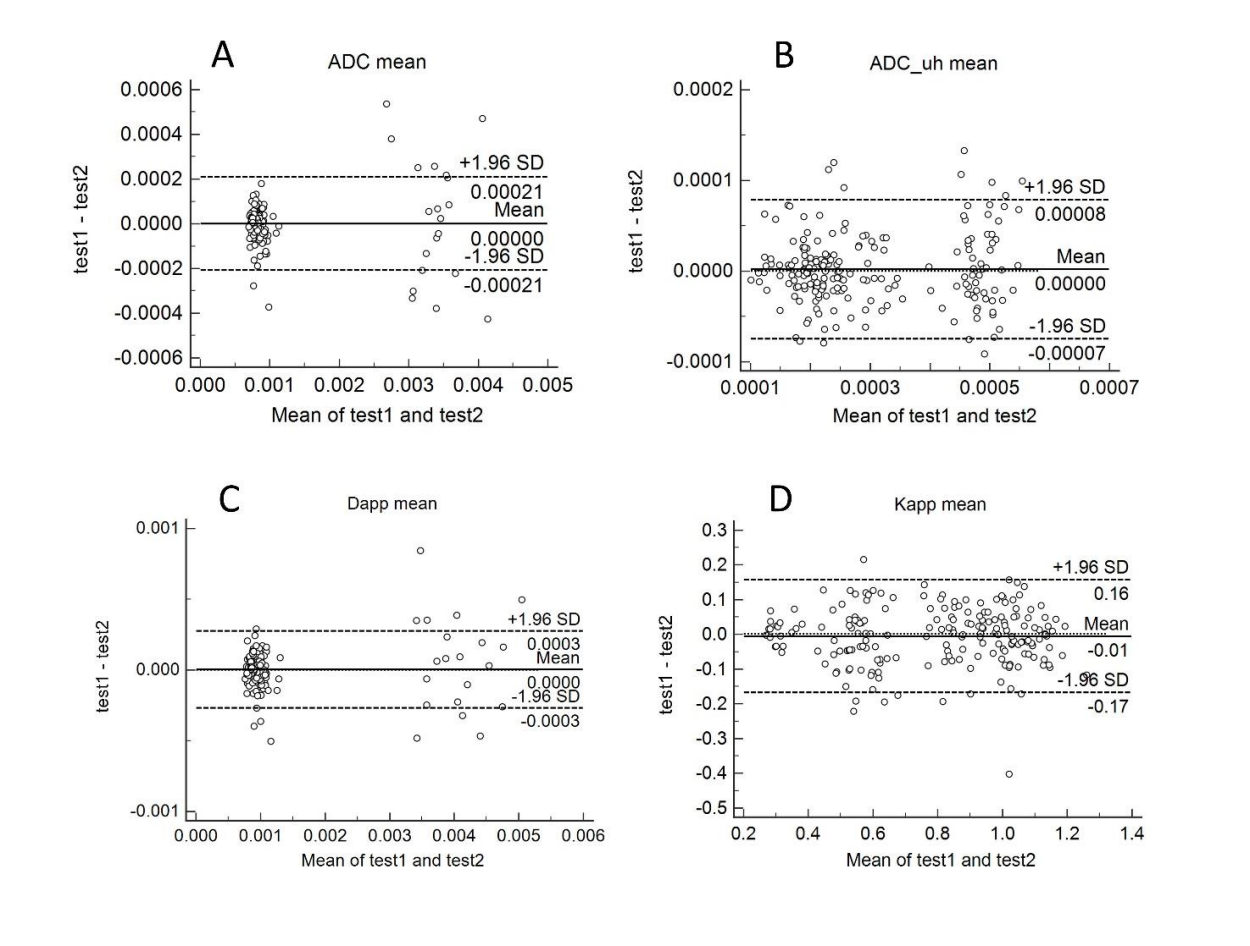
Bland-Altman plots of reproducibility of MRI. Bland-Altman plots for ADC mean (A), ADC_uh mean (B), D_app_ mean (C) and K_app_ mean (D) show a low mean difference between the two tests (continuous line: mean difference, dashed lines: 95% confidence interval of the mean difference).

In the repeatability test of the MRI, the mean and median values of all parameters are highly correlated (p<0.05 for all). For the kurtosis and skewness of ADC and K_app_, there is a good correlation between the two tests. However, for the kurtosis and skewness of ADC_uh, there is no significant correlation (Table 6). In the Bland-Altman analysis, there is no significant difference between the parameters of the two tests except for the K_app_ kurtosis (Fig. 3).

## DISCUSSION

It was originally found in our study that the ADC_uh values of deep WM in the bilateral TL of AD patients were higher than that of the HC group. This is inspired by an earlier study where the ADC value of averaged bilateral temporal stems of AD patients was reported to be higher than in healthy people [35]. Considering that the disease progression in each side of the brain may be asynchronous, we compared them separately in this study.

Compared to the HC group, the AD group showed increased ADC_uh values in the right CS. In the ADC_uh map, the signal intensity we measured is mainly due to the slow diffusion components, and AQP4 is a key part of the slow transport of water molecules in the brain. WM changes in AD patients include axonal damage and gliosis [36]. The distribution of AQP4 is closely related to astrocytes, as AQP4 is located mainly in astrocytic foot processes, particularly at the borders between the brain parenchyma and major fluid compartments [37, 38]. Another group of scholars found a correlation between ADC_uh parameters and the AQP4 expression in the solid parts of the cerebral astrocytoma [27]. Although no confirmation study of the changes in the distribution of AQP4 in the brain of AD patients was reported previously, we may assume that the changes in the ADC_uh map suggest some changes in the distribution of AQP4.

A previous study showed that the development of AD was closely related to the deposition of Aβ [39, 40], which exists in both the normal aging brain and the AD brain. The defect in the Aβ clearance may be the key reason for AD [37]. In the process of Aβ clearance, AQP4 plays an important role [21, 23]. Human amyloid precursor protein was overexpressed in HIP and the surrounding WM of transgenic AD mice [41]. This might be able to explain the changes in ADC_uh values in the white matter of bilateral TL in our study. In this study, we also found that the ADC_uh values in both the left THA and left LVe were significantly different between the AD and HC groups. The lateral ventricle contains a choroid plexus structure, its distribution in the ventricle is uneven, and an abundance of AQP4 was expressed in the choroid plexus [42, 43]. The changes of AQP4 expression have been confirmed in patients with AD [44], which might explain the changes in ADC_uh from the side.

**Table 4:** Correlations with MMSE score for all parameters.

	rho	p
ADC left FL _kurtosis_	550**	0.001
ADC right PL _mean_	-.368*	0.042
ADC right PL _median_	-.356*	0.049
ADC_uh right TL _mean_	-.420*	0.019
ADC_uh right TL _median_	-.386*	0.032
D_app_ left FL _kurtosis_	.546**	0.001
D_app_ right PL _mean_	-.416*	0.020
D_app_ right PL _median_	-.403*	0.024

FL, Frontal lobe; TL, Temporal lobe; PL, Parietal lobe. ADC and DKI have similar correlation with MMSE, and their locations are right PL and left FL. ADC_uh shows a special sensitivity to the correlation between right TL and MMSE.

A previous study of 7 pairs of samples, for all 3 b values =1000, 2000, 4000 s/mm^2^, reported that the mean diffusions in the regions of PL WM were higher in the AD group than in the HC group [28]. However, we found some differences only in the right TL WM between AD patients and HC patients in ADC maps. The sample size is different between our study and theirs. Their PL ROIs were placed in the subcortical WM in the PL, while our PL ROIs were placed in the deep WM. Those differences may contribute to the result above. The ADC value in the right HIP of our AD group was higher than that of HC group. This conclusion was similar to the results of other teams [35, 45].

**Table 5:** Comparison of receiver-operating characteristic (ROC) curves.

	AUC	95% CI ^a^	P^b^
ADC	0.826	0.694 - 0.918	NL
ADC_uh	0.766	0.627 - 0.873	0.501
DKI	0.847	0.718 - 0.932	0.728
ADC+ADC_uh	0.897^#^	0.779 - 0.964	0.172
ADC+DKI	0.840	0.711 - 0.928	0.729
ADC_uh+DKI	0.894	0.775 - 0.962	0.284
ADC+ADC_uh+DKI	0.868	0.743 - 0.946	0.416

AUC, the areas below the ROC curves.

^a^ Binomial precision;

^b^ Compared with ADC;

^#^ P=0.022 versus ADC_uh.

In our study, except for the right TL and left CS, all analyzed deep WM regions of AD patients showed abnormalities in the DKI parameters. The WM regions showed that the increased D_app_ and decreased K_app_ in this study were in agreement with the areas reported in some of the previous DKI studies [32, 46]. In this DKI computing model, the D_app_ values tend to be close to the ADC values [33]. In this study, abnormal regions on the D_app_ parameter maps are also found to be abnormal on the ADC parameter maps. The K_app_ parameter maps performed a high sensitivity for the WM of AD patients. A previous study has shown abnormal changes in DKI in cortical and deep gray matter of AD patients [47], which may be related to the difference in the choice of DKI related parameters. In our study, we only selected the parameters of D_app_ and K_app_. Of course, DKI can detect changes in the microstructures of the brain in patients with AD, include the intracerebral lesions caused by Aβ [48, 49]. Our cohort of patients included moderate AD patients. Compared with early AD patients, they may have more significant changes in the microstructure of brain. Thus, in our study, we can observe several brain regions with abnormal changes. More lesions on DKI than ADC or ADC_uh can be observed, although it cannot include all the abnormal areas of ADC or ADC_uh. K_app_ of bilateral LVes have shown abnormalities, which may be related to an uneven choroid plexus distribution in bilateral LVes. Several studies have shown significant changes in the histological morphology of choroid plexus in AD patients [50, 51].

**Table 6:** The correlations of ADC, ADC_uh, D_app_ and K_app_ parameters between the two tests.

	rho	p
ADC mean	0.782	0.000**
ADC median	0.760	0.000**
ADC skewness	0.194	0.006*
ADC kurtosis	0.226	0.001*
ADC_uh mean	0.901	0.000**
ADC_uh median	0.897	0.000**
ADC_uh skewness	0.133	0.061
ADC_uh kurtosis	0.058	0.412
D_app_ mean	0.710	0.000**
D_app_ median	0.675	0.000**
D_app_ skewness	0.169	0.017*
D_app_ kurtosis	0.142	0.046*
K_app_ mean	0.929	0.000**
K_app_ median	0.934	0.000**
K_app_ skewness	0.193	0.006*
K_app_ kurtosis	0.278	0.000**

^**^p<0.001,

^*^p<0.05; Spearman’s rank correlation was used.

The correlation analysis showed that the brain areas that correlated with MMSE in ADC and DKI were consistent, while ADC_uh of the right TL have a negative correlation with MMSE. The result of the correlation analysis also suggests the unique sensitivity of ADC_uh to TL WM lesions. In a previous study, the resting state functional magnetic resonance imaging (rs-fMRI) was used for AD classification. The AUC of a single parameter was 0.82-0.84, and the AUC of a combination of multiple parameters was 0.85 [52]. In our study, ADC and DKI independently showed a similar ability of classification. When they were combined with ADC_uh, increased AUC values were obtained. However, the performance of the combination does not differ significantly from ADC. Currently, the change of cortical gray matter volume is still one of the most sensitive indices of AD. Some scholars have classified AD from controls by using Voxel-wise gray matter densities and achieved a highest AUC of 0.941 in their research [53].

There are several limitations in our study. First, a previous study found that amyloid aggregation in the brain of AD was not linearly related to the progress of AD [54]. Our cohort did not group early and medium AD patients separately, which may have an impact on our results. Second, the number of cases involved in the study is small. The age of the AD and HC groups is different, and the age span is too large. Third, the slice of the MRI image is thicker, there are some small structures such as HIP that cannot be completely shown, and the selected ROI may be the body or head. Finally, the degree of WM degeneration may also be affected by education, work, or basic diseases such as diabetes, hypertension, etc. The effects of these factors were not corrected in this study.

In summary, the detectability of AD by ADC_uh does not differ significantly from that by ADC or DKI. However, ADC_uh combined with ADC or DKI can obtain a higher AUC value. ADC_uh can reflect some special physiological and pathological changes of WM in the unique regions of AD brain, which have not yet been clearly revealed, and AQP4 may be an important part of them. This characteristic is obviously different from those of ADC and DKI. Additionally, the utilization of ADC_uh is potentially useful for noninvasively monitoring the pathophysiological changes of AD and the diagnosis of AD.
